# S156. FRONTO-STRIATAL FUNCTIONAL CONNECTIVITY AND STRIATAL DOPAMINE CAPACITY IN TREATMENT-RESPONSIVE AND REFRACTORY SCHIZOPHRENIA

**DOI:** 10.1093/schbul/sby018.943

**Published:** 2018-04-01

**Authors:** Seoyoung Kim, Wi Hoon Jung, Euitae Kim, Jun Soo Kwon

**Affiliations:** 1 Seoul National University Bundang Hospital; 2 Korea University; 3 Seoul National University College of Medicine

## Abstract

**Background:**

Schizophrenia is thought to be a heterogeneous disorder and evidences reflect categorically distinct subtypes according to the antipsychotic treatment response. Altered frontostriatal functional connectivity (FC) in schizophrenia and its correlation with antipsychotic treatment response also suggests divergence of underlying pathophysiologic mechanism. Meanwhile, the observations that prefrontal activity correlates with striatal dopaminergic function, leads to the hypothesis that the disrupted frontostriatal FC would be related with altered dopaminergic pathway in schizophrenia. The aim of this study was to investigate the relationship between frontostriatal FC and striatal dopaminergic activity in patients with schizophrenia according to the responsiveness to first-line antipsychotic drug.

**Methods:**

24 symptomatically stable schizophrenia patients were recruited from Seoul National University Hospital, 12 of which responded to first-line antipsychotic drugs (first-line AP group) and 12 stable under clozapine (clozapine group), along with 12 matched health controls. All participants underwent resting-state functional MRI and [F18]DOPA positron emission tomography.

**Results:**

There were no significant difference in the total PANSS score between the first-line AP group and the clozapine group (mean difference=0.67, s.e.=3.21, df=33, p=1.000). Voxel-based analysis found significant negative correlation between frontal FC to the left associative striatum and the kicer in the corresponding region was found in first-line AP group but not in clozapine group or healthy control. Additional region of interest analysis confirmed the result (control group: R2=0.032, p=0.572; first-line AP group: R2=0.551, p=0.005; clozapine group: R2=0.108, p=0.297).

**Discussion:**

Different patterns of relationship between striatal dopamine capacity and frontostriatal FC observed in this study indicates different pathophysiology underlying schizophrenia according to antipsychotics treatment-responsiveness. Results should be reconfirmed in prospective manner with larger sample size in future studies.

